# Back to the Future—Moving Forward for Testicular Cancer Survivors

**DOI:** 10.1093/jncics/pkz082

**Published:** 2019-10-08

**Authors:** Craig R Nichols, Lucia Nappi, Christian Kollmannsberger, Robert Hamilton, Siamak Daneshmand

**Affiliations:** 1 Testicular Cancer Commons, Portland, OR; 2 British Columbia Cancer, Division of Medical Oncology, Vancouver, British Columbia, Canada; 3 Department of Surgery (Urology), Princess Margaret Cancer Centre, Toronto, Ontario, Canada; 4 USC/Norris Comprehensive Cancer Center, Institute of Urology, Los Angeles, CA; 5 SWOG Group Chair’s Office, Portland, OR

Agrawal et al. (1) characterize the magnitude of adverse health outcomes among US testicular cancer survivors as well as characterize modifiable risk factors. This study is an important output of the Platinum study under the direction of Lois Travis, now at Indiana University, from which these single institutional results are derived (2). These results expand the studies from European colleagues with an emphasis on actionable settings to reduce the severity of late effects attributable to cisplatin, etoposide, and bleomycin commonly used in the management of germ cell tumors (GCTs) (avoidance of high-decibel noise exposure, early diagnosis and management of metabolic syndrome, healthy lifestyles, avoidance of smoking). This study and others from Fossa and additional Scandinavian authors tell a critically important but fundamentally simple story. Cisplatin-based chemotherapy or therapeutic radiation for GCTs results in clinically significant adverse health outcomes in both the short term and long term in a number of survivors of GCTs. The incidence and severity of the adverse outcomes are largely related to a cumulative dose of chemotherapy, and these adverse outcomes can become more apparent over time. Although the severity of some of the late effects can potentially be modified, the reduction of the magnitude of the severity is far from complete, and in some settings, there are no known ways to modify the risk (eg, therapy-related malignancies, development of metabolic syndrome, neuropathy).

Unfortunately, these important data are often greeted in the medical oncology or radiation therapy communities with a tacit acceptance that adverse health outcomes are part of the “cost” of a reliable cure of metastatic cancer and that most late effects occur “out of sight and out of mind,” often after care transition from the GCT care providers complicates awareness and interventions.

Consider survivorship in GCTs as an inverted funnel where patients enter at the time of diagnosis with or without subsequent chemotherapy, extensive surgery, or radiation therapy. In the United States and Canada, approximately 11 000 patients are diagnosed with GCTs annually. After a few years of either observation or definitive treatment, about 10 500 of these patients will enter the large and expanding portion of the funnel where they will remain for the rest of their lives. An estimated quarter million survivors of testicular cancer and its management live in North America with a per-patient additional life-years added of 40–50 years postdiagnosis—not a small challenge by any means.

Let’s think about the large proportion of patients who are posttreatment, which is the same group addressed by the Agrawal study (1). Currently, their adverse health outcomes are mostly baked in. We can’t go back and unring the chemotherapy, radiation, or surgery bell. Dissemination of customized information regarding late risks and modifiable risk factors is very difficult for this far-flung population. Implementation of survivorship care plans and transition of care plans is extremely uneven. For GCTs, general oncology and primary care providers do not consistently provide optimal, personalized, and timely discussions of risks of late effects, nor do they consistently counsel on the potential salubrious lifestyles to minimize late effects.

Little recent attention with respect to survivorship has been paid at the narrow end of the inverted funnel (ie, when upfront curative treatment decisions are being made). The recent history of academic GCT oncology has not concentrated on delivering similar high cure rates with less chemotherapy, less radiation, or less imaging, all of which are fundamental drivers of short-term and late effects and patient bother. Current practice and clinical investigations have often been pointed to more toxic approaches; combined approaches; and exuberant, unnecessary imaging (3–6).

Successful delivery of newly diagnosed patients to the other side of survivorship should entail meaningful reduction in the number of cycles of chemotherapy delivered, elimination of prophylactic and therapeutic radiation therapy, and a marked reduction in imaging. Two emerging themes in GCT clinical research oncology offer hope of achieving this goal: precision medicine and centralized and collaborative clinical decision support and triaging complex care to high-volume centers.

The most exciting development in precision medicine in GCTs comes from two recent, large prospective studies with serial measurement of serum or plasma microRNA 371 (7,8). These studies demonstrate that this biomarker has very high specificity and positive predictive value to a degree that it appears likely to someday be able to reduce the use of computed tomography scanning in active surveillance and postchemotherapy imaging. In addition, miR371 has the potential to drive down disease volumes at which GCT relapse is discovered while on active surveillance.

Primary retroperitoneal lymph node dissection (RPLND) is somewhat limited in large part because of the insensitivity of current imaging and classic marker-based determination of relapse (up to 30% false positive in expert centers). Since the 1970s, primary RPLND alone in the common setting of small-volume, marker negative stage II nonseminoma is known to be remarkably effective without the need for adjuvant chemotherapy (relapse free rate of approximately 75%). Several centers in the United States and Europe are testing an identical approach in small-volume stage II seminoma with similar preliminary results seen when classic, open primary RPLND is applied (9–11).

If clinical trials using miR371-guided and expert surgical management of early stage GCT demonstrate equivalent therapeutic outcome with reduction or elimination of the use of radiation-based, chemotherapy-based approaches and computed tomography–based imaging, nearly 85% of patients presenting with any stage of GCT will enjoy those subsequent 40 years with nearly zero risk of severe late effects of treatment or frequent imaging. These innovations along with increased focus directed at chemotherapy-sparing and imaging-sparing approaches will likely further reduce the number of GCT patients at risk for consequential late effects of treatment. No small victory by any means.

## Notes

Dr. Lucia Nappi, Honoraria: Astellas Pharma, Pfizer. Patents, Royalties, Other Intellectual Property: Ivermectin analogue compound therapeutics for heat shock protein-27 (HSP27) inhibition, methods and uses associated therewith provisional No.: 62/756,707. Travel, Accommodations, Expenses: Pfizer.
